# Self-directed Science Learning During COVID-19 and Beyond

**DOI:** 10.1007/s10956-021-09953-w

**Published:** 2021-12-30

**Authors:** Libby Gerard, Korah Wiley, Angela Haydel Debarger, Sarah Bichler, Allison Bradford, Marcia C. Linn

**Affiliations:** 1 Graduate School of Education, University of California, 2121 Berkeley Way, CA 94720 Berkeley, USA; 2 Learning Sciences Research, Digital Promise Global, San Mateo, USA; 3 The William and Flora Hewlett Foundation, Menlo Park, CA USA

**Keywords:** Self-directed learning, Open education resources (OERs), Knowledge integration, Co-design, Research practice partnership (RPP)

## Abstract

Prompted by the sudden shift to remote instruction in March 2020 brought on by the COVID-19 pandemic, teachers explored online resources to support their students learning from home. We report on how twelve teachers identified and creatively leveraged open educational resources (OERs) and practices to facilitate self-directed science learning. Based on interviews and logged data, we illustrate how teachers’ use of OER starkly differed from the typical uses of technology for transmitting information or increasing productivity. These experiences provide insights into ways teachers and professional developers can take advantage of OER to promote self-directed learning when in-person instruction resumes.

## Self-Directed Science Learning During COVID-19 and Beyond

“It’s the blind leading the blind. We are on all these meetings about how to do Zoom, Google hangouts … but there is no cohesiveness. My principal is wonderful, but it is nebulous,” remarked a middle school teacher after California’s shelter-in-place mandate in March 2020. There was widespread concern among teachers as they rapidly transitioned from classroom to remote instruction. Wanting to retain compelling science learning experiences such as conducting experiments, resolving conundrums in data sets, and discussing alternatives with peers, teachers turned to technology. However, they soon realized that to offset the in-class guidance and peer collaboration that typically sustained student engagement, their students would need self-directed learning capabilities. We report on how twelve teachers identified and creatively leveraged customizable, scaffolded, and interactive open educational resources (OERs) to implement remote science instruction. OERs are teaching, learning, and research materials that are either (a) in the public domain or (b) licensed in a manner that provides everyone with free and perpetual permission to engage in the 5R activities—retaining, remixing, revising, reusing, and redistributing the resources (Creative Commons, 2021; Hilton, 2020). While OERs have the potential to foster students’ self-directed learning, they are rarely used for this purpose across US classrooms and especially in science (Cuban, 2018; Hilton, 2016). The ways in which these teachers leveraged OER for remote instruction has implications for how OER may be used to facilitate student-led science education for remote or in-person instruction.

### Self-directed Learning and Instructional Technologies

Self-directed learning is characterized by taking responsibility to reflect on one’s understanding, identifying gaps or questions, determining what resources will help one progress, and pursuing those resources to improve one’s understanding (Azevedo & Hadwin, 2005; Hmelo Silver, 2004; Kyza, 2009). While most current computer-based materials take little advantage of the power of technology, substantial research documents the effectiveness of some OER to invite new avenues for teacher agency, and for student-driven creation and exploration in science (Miller et al., 2021). OERs featuring scientific models, collaborative activities, and independent data exploration have shown success in guiding student knowledge integration in middle and high school science (Donnelly et al., 2014; Linn & Eylon, 2011) and in developing self-directed science learners (Hardy et al., 2019; Lee et al., 2019).

Some OERs that support self-directed science learning are standalone tools such as interactive models and virtual experiments developed by Phet, Chem Collective and others (Wilkerson-Jerde et al., 2015; De Jong, 2019), whereas other web-based platforms amalgamate multiple OERs to create lessons such as *Concord Consortium* (Hardy et al., 2019), the Web-based Inquiry Science Environment (*WISE)* (Ulus & Oner, 2020; Williams et al., 2012), and *nQuire* (Sharples et al., 2015). These OERs include research-based features that foster teacher and student agency, consistent with the goals of open education practices (Bali et al., 2020; Hilton, 2020). They encourage students to play with data to generate new ideas, explore alternative hypotheses, or test and revise explanations of phenomenon. They enable teachers to adapt instruction to meet their goals and students’ needs, such as by using logged student work or observations of student collaboration to inform their next step in guiding understanding.

Even with the widespread access to networked computers in American schools and the distribution of computers in response to COVID-19, most schools use OER to implement materials that emphasize transmission of information (Cambridge, 2018; Johnson, 2020). Such practices are in line with the U.S. Department of Education view that OERs serve as productivity and cost-saving tools: “the use of OER and other technologies can increase educational productivity by accelerating the rate of learning; reducing costs associated with instructional materials or program delivery; and better utilizing teacher time” (U.S. Department of Education, n.d. para. 2). Rather than supporting self-directed learning, many use OER to measure student retention of facts; display videos of concepts; or offer online versions of textbooks produced by major publishers (Cuban, 2018; Davis, 2016). This approach has largely failed students (Ahn & McEachin, 2017; Fitzpatrick et al., 2020).

In this study, we took advantage of a natural experiment brought on by the COVID-19 shift to remote instruction in which teachers selected, adapted, and facilitated OER to extend their in-person science teaching practices to the virtual space. We examine how a sample of twelve middle school science teachers who have a range of prior experience teaching with technology, and whose students reflect the demographics of students across the state of California, used OER for instruction during the first two months of the COVID-19 pandemic.

## Method

### Research Questions

We examine (a) how do teachers use OER in remote science instruction to promote knowledge integration, and (b) what are student perspectives on the use of OER for remote science learning? More specifically we examine what features of the teacher-selected OER are most valuable to teachers and students and why; what obstacles do teachers and students encounter in using this OER?

### Participants

When California schools went remote, many teachers located the *Web-Based Inquiry Science Environment (WISE)* OER for their instruction. The number of help requests received via the WISE contact page increased 40% with the shift to remote instruction as district curriculum administrators, science department chairs, and teachers sought assistance in planning. To respond, the WISE research group created weekly drop-in office hours, advertising it through the WISE website and emails to public schools in the region.

From the teachers who contacted WISE about using it for remote instruction, we then selected 12 teachers to study in depth who (a) had a range of experience with the WISE OER; (b) worked in schools serving different student demographics; (c) within a school, taught different grade levels; and (d) agreed to be added to our IRB in time to conduct interviews prior to the end of term. Six of the twelve teachers, as shown in gray shading in Table 1, started using the WISE OER for the first time upon the shelter-in-place mandate. The other six had used WISE at least once prior. Participating teachers taught at eight public secondary schools across the state of California. Five of the eight schools serve populations in which 65% of students are eligible to receive a free or reduced-price lunch; six of the eight schools serve a majority of non-White students. These demographics reflect the racial and economic demographics of public schools state-wide (60% receiving free/reduced lunch; 79% non-White). Students of six of the participating teachers (*N* = 330 students) who had agreed to be added to our IRB responded to the survey (Table 2). Although the units included posttests, the teachers often omitted them because grading was not required during remote instruction due to variation in student support at home.

### Curriculum

The teachers in this study selected one or more WISE units that feature interactive science models, data analysis tools, and discussion tools (Slotta & Linn, 2009). WISE is a platform that allows designers and teachers to integrate and adapt OER created by varied developers for their students. Specifically, the platform includes an authoring system that enables (a) designers to incorporate, sequence, and scaffold OER from other sources (e.g., Phet, Concord Consortium, USGS) and (b) teachers to modify unit content without learning programming, thus enabling teacher-driven customization.

The web-based units are aligned with the K-12 national science standards (NGSS Lead States, 2013). They are designed based on the constructivist knowledge integration (KI) pedagogy (Davis, 2003; Kali, 2006; Linn & Eylon, 2011). Emerging from longitudinal and case studies demonstrating that students develop and hold multiple, often conflicting, ideas about scientific phenomena (Clark, D’Angelo, & Schleigh, 2011; diSessa, 1988), the KI pedagogy helps learners take responsibility for constructing their own, coherent, evidence-based understanding (Linn et al., 2000). Specifically, units designed based on the KI pedagogy foster self-directed learning by enabling students to (a) articulate and build on their initial ideas; (b) discover new scientific ideas and evidence by interacting with peers, scientific models, conducting experiments; (c) use evidence or discussion with peers to distinguish among newly discovered ideas and their initial ideas; and (d) reflect on and connect ideas to form explanations.

### Data Sources

#### Teacher Interviews

We conducted a 30- to 45-min interview with each of the twelve teachers after they taught a WISE unit during the remote instruction period. The interviews asked about their transition to remote learning, their strategies to utilize the WISE unit and authoring tools to foster self-directed learning, and their reflections on the remote instruction experience. Two of the twelve teachers participated in two interviews (one after each WISE unit they taught during Spring remote instruction, responding to the same interview questions in each); ten participated in one interview (Table 2). Sample questions included the following: What changes did you make to this unit for remote instruction? Why did you decide to make this customization(s)? How did you facilitate learning in the unit — for example, did you hold any live office hours or communicate via email or phone with students? What do you notice about your students’ learning in the unit compared to when students are learning in the classroom? What part of this unit did you notice was most engaging and why? What did you observe was challenging for students during the unit and in what ways? What would you do differently next time when teaching this unit? Interviews were recorded and transcribed.

#### Office Hours

We conducted weekly, drop-in office hours on Zoom to support teachers’ planning for use of the WISE OER in remote instruction. The office hours Zoom link and weekly time were publicized on a website and through emails to public school teachers in the region. If a teacher consented to the IRB protocol, the researcher recorded the office hours discussion and took detailed notes. Seven of the twelve teachers participated in recorded office hours at least once for 30–60 min; two teachers participated in two office hours sessions devoting one session related to each unit they planned to teach. The majority of the teachers who used the office hours (5 out of 7) were teachers who had taught a WISE unit prior to remote instruction. The office hours discussions focused mainly on how the teacher wanted to customize a unit for remote instruction.

#### Student Survey

An end-of-unit student survey regarding the student’s perspective on learning at home was administered to students who were in schools that were included in the authors’ Human Subjects Protocol.

### Data Analysis

#### Teacher Interviews and Office Hours Field Notes

We used emergent thematic coding (Gibbs, 2007) to analyze the interview corpus. One author coded two interview transcripts, selecting one from a teacher with multiple years of WISE experience and one from a teacher new to WISE at the time of the study in order to establish the first set of themes. This was to ensure that the initial themes captured the range of teachers’ instructional approaches during remote instruction, across levels of teacher prior experience with WISE. The first set of emergent themes from this round of coding included some themes that aligned with the interview question foci (e.g., customizations, challenges), as well as new themes that emerged from the teachers’ responses (e.g., use of logged data to give feedback, interleaving WISE with other OER). The first author explained the themes with examples from the data to a co-author. The two authors then coded the same two interview transcripts separately and compared their coding. We compared: Did we code the same themes as present in each interview transcript, with having selected the same segment from the interview as evidence of the theme? After the first round of coding, we reached 65% agreement, and discussed disagreements and elaborated the description of the themes, as shown in Table 3. We repeated this process with two more interviews and reached 81% agreement. We discussed the disagreements and elaborated the description of the themes once more. One author then used this final set of themes, as shown in Table 3, to code the remaining 12 teacher interview transcripts. The field notes from the office hours were used in conjunction with the interview data to inform coding of if and how teachers customized the units.

**Table 1:**
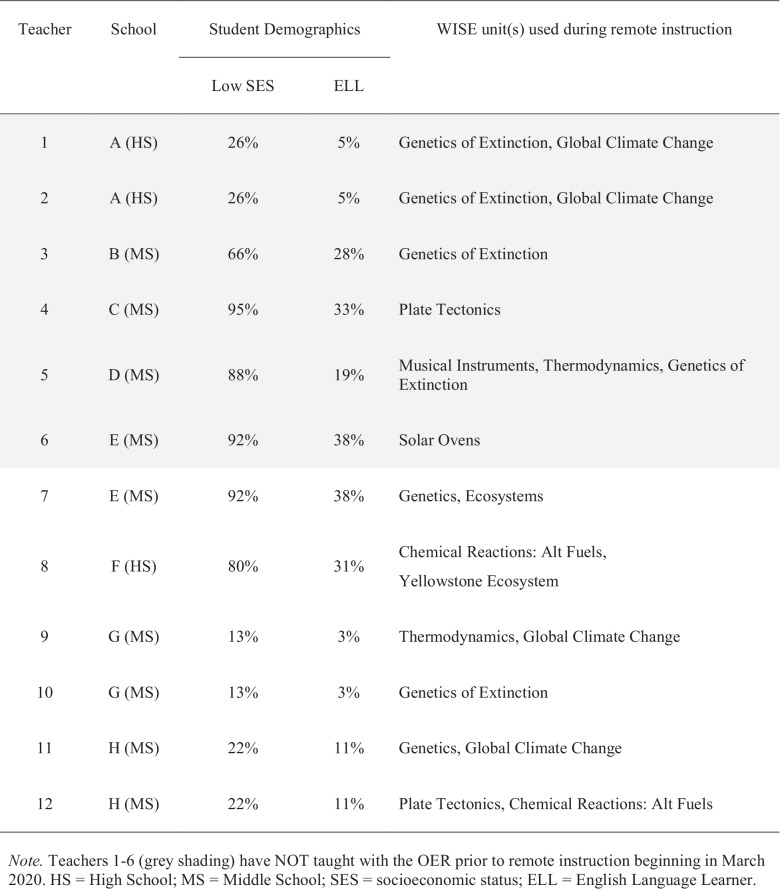
Teacher participants

#### Student Survey Analysis

The logged student selections and open responses were exported and aggregated across each unit. Student responses to the multiple-choice items are reported by frequency of student selection. For the open response item, the student answers were examined to identify common themes among the responses. Student quotes were selected that illustrated the most common themes.

## Findings

### Navigating the Landscape

All twelve teachers reported little guidance on how to shift to remote instruction when the shelter-in-place mandate occurred and they faced many difficulties. One teacher stated, “I am not a permanent teacher. I had only known my students for 3 weeks. It was really tough.” Another teacher, sick with COVID-19 as the school transitioned, had few resources. Many teachers noted the lapse in instruction as their district worked to ensure all students had computer and network access. “I work with a population of students who don't all have Internet access or computers so when we shut down so quickly all the students had was a Physical Science textbook—and we use the Integrated Earth/Life/Physical science model. So we had to…something random out of the book for three weeks [until] the district had given computers and Internet access to all students who needed it, [then] we started WISE.” One of the teachers from a large district in Southern California shared, “There was a lot of low quality stuff put out there, a lot of drill and kill stuff. …I was trying to figure out how to do inquiry science in the distance environment when we had a couple of days ahead to prepare. All students have a chromebook and they provide hotspots to kids without internet… So day before school shut down I had everyone log on and create a WISE account and start the Sound unit.”

### Teachers Leverage WISE OER for Self-directed Learning

**Fig. 1:**
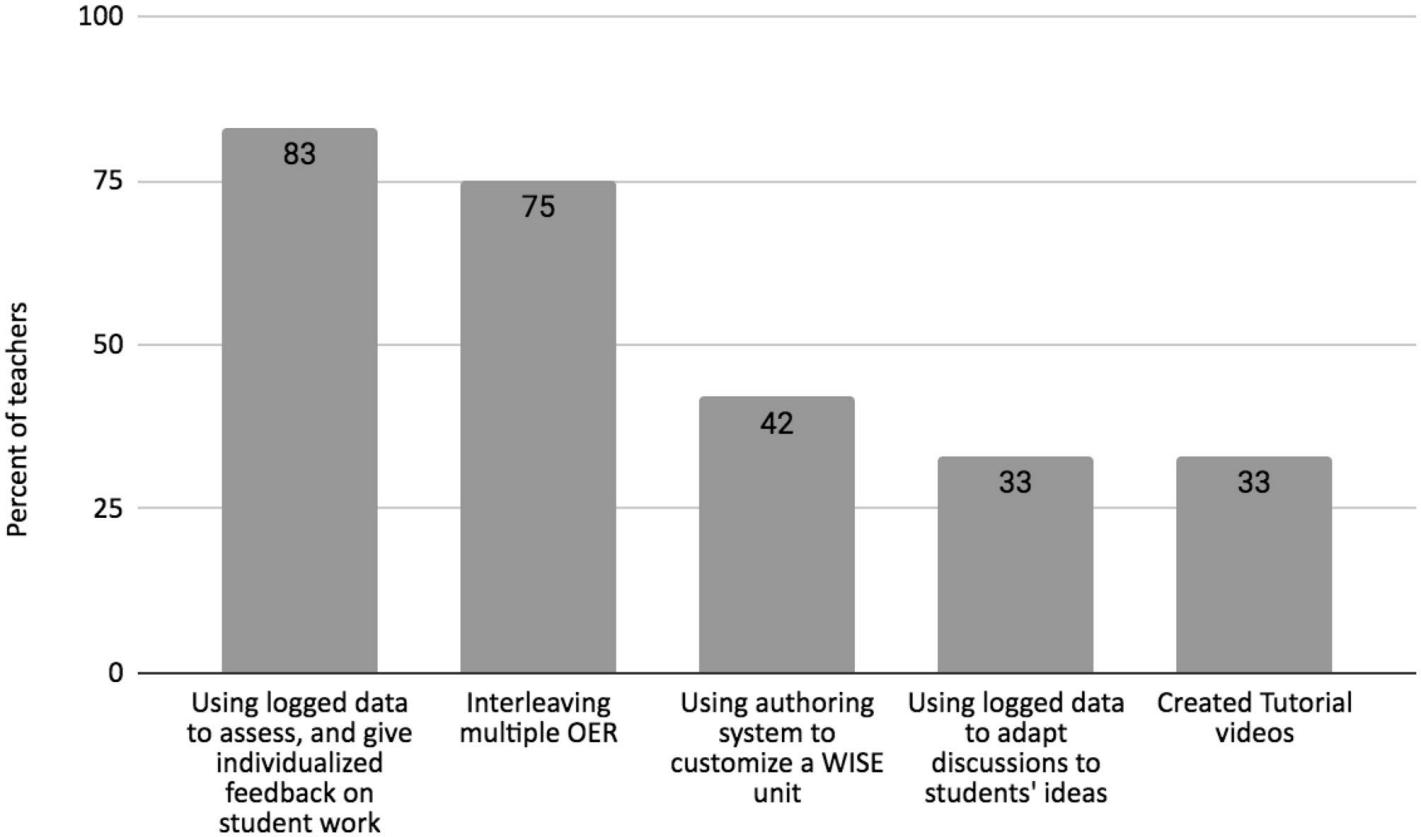
Teacher strategies to facilitate self-directed learning with the WISE OER

We identified several distinct strategies that teachers created to facilitate students’ self-directed learning and to respond to identified student needs during remote instruction (Fig. 1).

### Using Logged Student Work and Automatically Scored Explanations

All but one of the teachers used the student work logged by the WISE system to capitalize on an unexpected opportunity for providing students with individualized support. As one teacher shared, “[Providing individualized instruction] was easier to manage through distance learning because then you're not really running in different directions at the same time [like in classroom instruction] and instead you are addressing individuals, giving each individual feedback.” Specifically, teachers used the logged student responses to write personalized guidance to each student (83%), to design class discussions in which they collaboratively examine individual student ideas or analyze common student ideas across the class (33%). Whereas almost all of the teachers reported that the logged student work helped them gain new insight into their students’ thinking, the teachers leveraged the logged data in different ways to craft individualized guidance (Table 4). For example, some valued seeing how individual students were distinguishing among their intuitive ideas about the science concept and the new ideas discovered in the unit. Others valued the opportunity to encourage their students at a distance, and some appreciated how the automated scores helped them efficiently target an intervention that responded to a student’s ideas.^1^

**Table 2:**
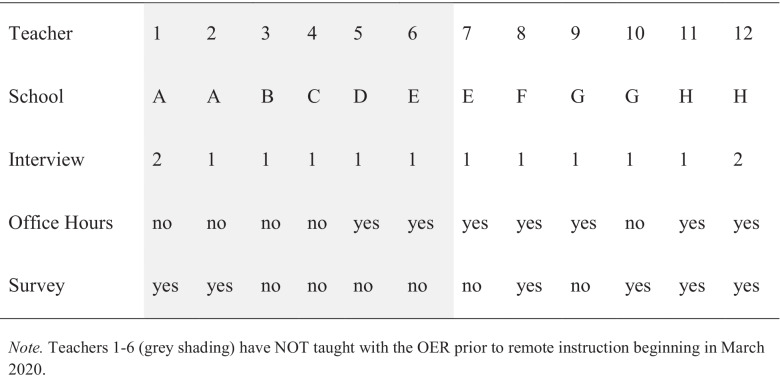
Data sources

### Interleaving WISE and Other OERs

When teaching with the WISE OER, nine of the twelve teachers (75%) interleaved other OER with the WISE unit to increase interactivity during synchronous instruction, and to provide additional evidence in response to what they observed in students’ science thinking. To increase interactivity during synchronous instruction, they used OER such as interactive Google Slides/PearDeck, a Kahoot game-based quiz, or digital flashcards. To enrich the scientific evidence, they added resources such as an open-source data set available on the Howard Hughes Medical Foundation, or the U.S. Department of Energy websites, a video from CK12.org, or a simulation from Phet Interactives. Nine of the 12 teachers (75%) used an open access learning management system, specifically Google Classroom, to distribute assignments and manage communication.

### Creating Tutorial Videos

To facilitate students’ self-directed learning at home, four of the 12 teachers (33%) developed videos of themselves explaining features they would have typically demonstrated in a face-to-face class. One teacher remarked, “Using screencast… I would show them what to do specifically in certain steps if I felt like it was more of a complicated step.” Another teacher explained, “I made guidance videos for how to use simulations, enable flash…I would read the steps…, that hand holding was more for my students with special needs or my ELL students or just students who had a hard time focusing…” By creating videos to provide clarifying instructions, teachers reduced the amount of time they spent responding to student emails about technical questions and increased the time they had available to interact with students on other aspects of instruction, such as giving feedback on students’ science explanations or addressing content-related questions.

### Customizing for Students’ Needs

Five of the 12 teachers (42%) took advantage of the adaptability of the WISE OER and the authoring system to adapt unit content to meet the specific needs of their students (Table 5). These five teachers were all teachers who had previously taught with the WISE OER and attended prior professional development utilizing the authoring system, prior to the March 2020 shift to remote instruction. They drew on their knowledge when the shift occurred, and three of the five joined office hours to discuss their customization plans with a WISE researcher. The customizations that these teachers made reduced technical challenges in the units. They also modified instructions to increase student agency in exploration. For example, one teacher reported how she modified the instructions for student experimentation with an alternative fuels computer model to encourage analysis of trade-offs: “Changed the third line of the instructions to ‘Use the model to find the optimal balance between carbon dioxide produced and monetary costs’ to keep their focus on efficiency instead of just playing with the model.”

**Table 3:** Teacher interview coding

Coding themes *italics = r*efinement made to the theme after coding round 1; refinement made ****after coding round 2***		**Round 1** **1 = agreed for both interviews; 0(X) = disagreed for 1 of the interviews or both**	**Round 2**
Teaching strategies with OER to facilitate self-directed learning	Interleaving other OER	0 (1)	1
	Tutorial videos	1	1
	Using logged data to assess learning, give individualized feedback on student work, have students revise	1	1
	Using logged data: using student work to personalize a group, individual or class discussion	1	1
	Using authoring system to customize the WISE unit	1	1
Teacher reported OER Features that Promoted self-directed learning	Students experiment with interactive models, test ideas in ***interactive activities***	1	0 (1)
	Students use guidance to evaluate and revise: *Reflecting on what they know, integrating evidence, reformulating understanding; ****monitoring their progress***	0 (both)	0 (1)
	Student led activity structures that motivate agency such as taking action in a societal issue; solve complex problems; engage in *multiple ways of learning such as* hands-on design, *writing, analyzing videos*	0 (both)	1
	Targets NGSS goals *or other science topic that was not covered by the school adopted science text, aligned with a topic they wanted or planned to teach*	0 (1)	0 (1)
Challenges of using OER for self-directed learning and suggested improvements	Low participation due to limited computers, internet, *or parental support, or encountered ****other technical issues***	0 (1)	1
	Assessment using logged data only, missed other indicators like discussion	0 (both)	0 (1)
	Student engagement, lack of motivation	0 (1)	1
	Need for just in time scaffolding*, in-person verbal clarification, or ****teacher guidance while working***	0 (1)	0 (1)

Other teachers customized the unit to strengthen support for students’ academic language development, implementing tested strategies like sentence starters. Still others updated the unit’s science content to address an additional concept (e.g., slab-pull as part of the mechanism in Plate Tectonics), or to connect the topic to their students’ lives (e.g., incorporating a reference to a school community member; examining a popular car company’s fuel plan).

### OER Features for Self-directed Learning

**Fig. 2:**
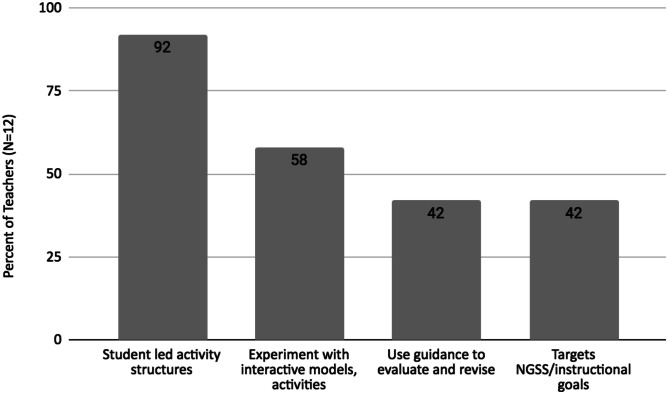
Teacher-reported features of WISE OER that promoted students’ self-directed learning

Teachers also reported features of the OER that they believed best supported students’ self-directed learning in the science classroom (Fig. 2).

Eleven of the twelve teachers (92%) appreciated how the student-led activity structures encouraged students to take ownership for constructing knowledge***.*** One teacher shared “Students liked how it started off with like what do you think, here's some information, revise your thoughts. They really like that kind of a [pedagogical] model.” They also valued how students could determine how to gain new knowledge based on their distinct learning needs. This included opportunities for students to take on a more challenging task than assigned. A teacher who taught Solar Ovens in a special needs class noted, “The students asked if they could go above and beyond [making smores in the oven] and make the temperature even higher so they could make something else. They saw the simplicity of the oven and saw they could do more with it.”

Teachers were particularly enthusiastic about how the activity designs encouraged students to apply their new scientific ideas to address a societal issue. A middle school teacher remarked “I liked teaching kids how you can write to your representative and tell them your opinion [about the tradeoffs between electric, gasoline and natural gas powered cars]. In the end of the unit, I had this great conversation with [a group] of girls, …they were skeptical that the governor was really going to read the letters they wrote [in the unit]. I explained to them that your letter does make a difference even if he does not specifically read it. They were convinced by the end that a teenager does make a difference. Overall it was a really good lesson that they are NOT powerless, giving them a sense of agency, power.” A high school teacher commented “I think they [students] were interested in what humans are doing… like in lesson 8, are humans causing global warming… that’s where we are engaging more people.”

Teachers observed that some students demonstrated agency when investigating a societal issue that teachers had not observed in the student’s behavior in the classroom. A high school teacher who taught a unit on the economics of alternative fuels commented: “One student told me, I wasn’t interested but I enjoyed doing it…It’s not a full spark but it’s a partial spark. That person found out that it was worthwhile to learn about things besides the normal stuff in the textbooks. That was a great unexpected outcome of this for me.”

### Experimentation with Interactive Scientific Models, Graphs, and Activities

Seven of the twelve teachers (58%) reported benefits of student experimentation with the interactive scientific models and graphs as a central way for all students to discover new ideas about science concepts. In particular, teachers valued students’ ability to gather data related to their conjectures about the concept. A teacher commented, “The most engaging part of the Climate Change unit was the Netlogo model, the different options, because they could try out different things and see the scale afterwards.” Some teachers appreciated how the models supported students in analyzing data to form their own perspective. A teacher noted “It’s more tinkering with models and judging the outcomes to select the best option. …a lot of opportunity for deep analysis. It makes them think.”

### Guidance for Revising Explanations

Five of the twelve teachers (42%) identified the embedded guidance, whether assigned by the teacher or computer, that prompts students to revise their work as key to their students’ knowledge building process. Teachers reported that requiring revision encouraged their students to hold themselves accountable to iteratively improving their understanding when the teacher is not physically present.

### Aligned with NGSS or Instructional Goals

Five of the 12 teachers (42%) reported that it was difficult to locate OER that aligned with the grade-level aligned NGSS performance expectations. They appreciated identifying an OER which addressed the topics identified by NGSS and used a pedagogy that engaged students in the NGSS identified science practices.

### Challenges of Using OER for Self-directed Learning

The primary challenge to using OER for promoting self-directed learning in remote science instruction was the inequities in students’ access to technology and the internet. Other challenges were similar to those in the classroom but were exacerbated by remote learning. Teachers had difficulty giving students the help they needed when they were stuck, similar to what they would have provided in the classroom such as nudging students who received guidance to revise; pushing students to persist when the content or instructions were perplexing; or proactively encouraging students who often need additional scaffolding. Eight of the 12 teachers (67%) reported an overall lack of student engagement during remote instruction compared to in-person instruction.

While the logged student work gave teachers insight into student thinking, two teachers lamented the limitations of interpreting student understanding from written text. One teacher noted missing the dialogue he would normally hear among students “When there are two students doing a WISE project in the classroom together there is a lot more discussion going on. I can walk around the room and answer questions, I can track them better.” Another teacher explained how relying only on students’ written explanations during remote instruction, particularly for those who are English Learners, does not reveal the students’ full understanding: “Sixty-something percent of my students are English Learner students and so their English language skills are low. I think that's just, it's kind of a limitation of the digital set up because of its reliance on students having, you know, good English language skills, because if the computer or I read it, I don’t have the context of the actual person.”

### Student Perspectives on Using an OER for Remote Instruction

Students echo their teachers in missing the just-in-time guidance and the motivational support provided by teachers and peers during typical in-class instruction (Table 6). Although many enjoyed working at their own pace during remote instruction, they wanted to work with a teacher and peers in-person, and desired more resources when exploring the science topic to clarify or deepen their investigation.

**Table 4:** Categories of teacher reported value of logged student work

Teacher reported value of logged student work	Representative teacher reflection [reported value emphasized]
**Makes individual’s scientific thinking visible**	
See how students are distinguishing among their ideas	“I’ve noticed in reading a lot of their responses that they’re not very good at using correct terminology about things. For instance, *I am seeing in their explanations about how lizards needed to adapt to the new situation… a lot of their answers imply that it's more of a Lamarckian evolution where they think lizards want to be faster.”*
See individual student’s thinking process	“*I really, really liked the window into students’ thinking* that WISE provided… I noticed I had a really clear idea of what students were thinking. *There was evidence for what they are actually thinking. It goes beyond them just getting the right answer… sometimes they would surprise me, provide an answer that was actually a good insight when I thought they did not understand.”*
**Enables teacher to comment, encourage student to persist**	
Encourage students to persist	“The teacher feedback piece is super valuable, just *because I’m not there to talk to them individually or talk to the whole class* and that was a feedback I got from my students is that *they were desperate for feedback, some kind of back and forth*”
Hold students accountable and give specific guidance	“Written responses allowed for easier teacher feedback and *helped hold students accountable. It allowed me to be specific with feedback* (full sentence, make a claim with evidence, etc.).”
**Has automated scores to support efficient teacher intervention**	
Efficient assignment of personalized guidance	*“I saw what area my students were proficient in [using the auto score] and then in which they needed improvement and my comments were trying to guide them* toward improvement. The information that the automated scores gave me was good to show the general pulse of what my students were thinking.”
Planning responsive class discussions	*“The [teacher report summarizing the automated scores for student explanations] gave me a really nice target for our discussion section because it was really clear what the major issue was across students and I could prepare for our discussion section based on that*. I think otherwise I probably would have conducted it in a more ad hoc fashion where I would have some general discussion questions and then whatever comes up, comes up.”

**Table 5:** Categories of customizations teachers made to the WISE units

	Teacher				
	1	2	3	4	5
**Improving learner access**					
Reducing technical challenges (e.g., clarifying navigation, blurry image)	**x**	**x**	**x**	**x**	**x**
Modifying text to clarify instructions (enumerating, simplifying text)	**x**	**x**	**x**	**x**	
Adding academic language supports into the unit (e.g., adding terms used in class, highlighting words, sentence starters, giving definitions)	**x**	**x**	**x**		
Modifying an activity structure in the unit to better align the scaffolding with their students’ needs (e.g., provide more structure, increase or reduce writing)	**x**	**x**			
**Supporting cognitive engagement**					
Expanding the unit to address additional content by incorporating new animations, and/or text	**x**	**x**	**x**		
Adding checkpoints into the unit to pace and monitor student progress			**x**		**x**
Incorporating previously successful classroom activities into the unit (e.g., google slides, drawings, videos)	**x**				
**Increasing relevance**					
Modifying activities to connect to students’ lives (e.g., location, political context, student interests, students’ family structures)	**x**	**x**		**x**	

Teacher 1 taught Chemical Reactions and Plate Tectonics; teacher 2 taught Ecosystems and Genetics; teacher 3 taught Chemical Reactions and Ecosystems; teacher 4 taught Genetics and Climate Change; teacher 5 taught Genetics of Extinction

**Table 6:** Student perspectives on learning with WISE OER at home

Question	*N*	Answer options	Frequency	Example responses [selected to illustrate the most frequently reported category]
What did you enjoy about doing this unit at home?^1^	330	Self-paced, flexible	81%	“I could do it on my own time without being told when I had to do it and how much I had to get done in that certain period of time.”
		Working individually	44%	
		Content	20%	
What was difficult about learning from this unit at home?^1^	305	Not having my teacher present	57%	“When there wasn’t a teacher usually the questions I had I would forget about before I had the chance to ask them.” “For me, I find it difficult to either ask a question on a scheduled call, or on an email. It is much easier to ask about any small questions when I am in class and I can ask my classmates around me or my teacher.” “It was difficult to find motivation without my teacher.”
		Content was difficult to understand on my own	40%	
		Not having my classmates present	38%	
		Finding space and time to work on the project	30%	
		Not working with a partner	30%	
What do you wish you could have had access to help you learn during this unit — write anything that you think would be helpful!^2^	305	Nothing	37%	“I actually think it was easier learning from home than it would have been learning this unit at school.” “I wish that there was an equation showing how much CO2 would be produced by a car running on electricity made by natural gas, and how that compares to coal.” “A video explaining the parts of an engine for electric cars and gasoline cars.” “I just think its easier when i can have a teacher to ask questions to in class and a partner to work with.” “I wish I would have been able to do this project with a partner since I would always learn more with help from a friend.”
		More resources to explore/explain the topic (e.g., videos, models, lab)	25%	
		Having a teacher or peer present	22%	
		Help when needed it/more directions	15%	
		Be in the classroom	4%	
		Specific changes to content	3%	
		Improve tech or home environment	3%	

^1^Multiple choice item, students could choose more than one response. ^2^Open response

## Limitations

The generalizability of the student survey results is limited to students who had access to the resources needed to participate in remote science instruction. The paper excludes analysis of student learning due to missing posttest data and likely bias. Some teachers omitted the posttest because grading was not required during COVID and others made the posttest optional. Teachers noted that analysis of learning would be biased towards students who had resources at home to support engagement in remote instruction.

## Discussion and Conclusions

The sudden shift to remote instruction showcases the value of the OER that support teachers in adapting to new circumstances. They responded to the remote learning challenges by facilitating student interaction with the OER and some adapted the OER to meet their students’ needs. In contrast to typical use of OER for transmitting information or increasing productivity, teachers used the OER in ways that interactively engaged students in expressing their science ideas, testing their conjectures using computer models, and revising their explanations in response to feedback. Their experiences provide insights into ways to support teacher use of OER to promote self-directed learning for both remote and in-person instruction.

### Teaching Practices with OER: Facilitating Self-directed Learning

Teachers who are both new and experienced in using OER can take advantage of the OER features to foster learner agency in secondary science education by adopting the technology-enhanced teaching practices exemplified by these teachers. First, teachers took advantage of logged student work to create class discussions and provide written feedback tailored to students’ ideas. Each of the teachers valued how the logs of student work, collected as students are engaged in the learning process, provided a unique window into their students’ range of ideas and alternative interpretations of evidence. It also allowed teachers to see how students refined their ideas in response to teacher guidance. Providing personalized and timely guidance was particularly salient for affirming students’ identities as science learners during a time when much of students’ school experience was occurring in isolation (Aschbacher et al., 2014).

A second teaching practice was leveraging the web-based authoring system of the OER to customize the curriculum content. The five teachers who had previously taught with the OER as a part of a WISE Research Practice Partnership (RPP) used the web-based authoring system to modify activities. They removed technical obstacles, deepened opportunities for student exploration of content, helped connect science content to their students’ lives, and scaffolded the use of text to support students’ academic language development. These customizations demonstrate the value of RPPs in supporting and empowering teachers to adapt OER to meet their students’ needs.

The experiences of these teachers help us identify the professional development support future teachers may need as schools take up OER. Future users of OER will benefit from the ability to take advantage of the authoring capabilities to customize their instruction. In particular, future professional learning may support teachers to customize OER to increase student access to materials, scaffold cognitive engagement as students progress through a unit, and to localize the curriculum to their students’ environment. OER developers may be more mindful of creating authoring tools that enable users, particularly those without programming experience, to make modifications. As seen in this study, RPPs can support the mutual benefits of OER customization. RPPs may assist teachers in aligning their curriculum customizations with pedagogical goals and leveraging technology advances. The teachers led the customization process in response to COVID-19, giving the researchers insights into the features of materials that strengthen support for students’ self-directed learning.

Other teaching practices that leveraged OER were facilitating students’ use of interactive models and activities to test conjectures about the science and guiding students to iteratively refine their explanations based on evidence or guidance. Teachers welcomed the opportunity during remote instruction to give students ample time to self-direct their exploration of a virtual model, testing variables and reformulating their explanation of the phenomenon. Students likewise appreciated the ability to explore the models and construct their understanding at their own pace. In typical instruction, teachers often feel pressured to limit students’ self-directed exploration with models or to revisit and revise, due to the pressure to “do school” (Russ & Breeland, 2019). This study reinforces the value from both the teacher and student perspectives of creating space and guidance for students to take ownership in making conjectures, exploring their ideas, and gathering feedback on emergent understanding.

Setting goals for student progress in a guided lesson, and encouraging students to self-pace towards that goal remains possible during in-person instruction. To support this approach in person, teachers suggested for example strategically partnering students so they are able to help each other with both content and pacing. Additionally teachers may guide students who work more quickly to help their peers who are feeling stuck, or allow students to work individually but group students physically together so they can still collaborate and seek peer feedback. Teachers can use logged student work to facilitate the grouping of students who are working on the same part of the lesson or could mutually support each other in different activities.

### Value of Well-Designed OER

The teaching practices showcased here point to the need to identify and develop well-designed OER to support all students in directing their science learning. The teachers echoed the need for materials that supported each student to learn. Science instruction often involves a reliance on text to access and communicate ideas, limiting some students’ methods for learning and expressing themselves (Lee, Quinn, & Valdez, 2013). However, by leveraging OER that promoted inquiry with scientific models, students were able to use diagrams and graphs as avenues for expressing their understanding. Beyond remote learning, OER features that facilitate peer dialogue and joint exploration of dynamic models can encourage the type of idea expression and exchange that are critical for learning and assessment (Boda et al., 2021; Ryoo & Bedell, 2019).

Further, OER features gave students flexibility in controlling their pace through the units. Letting students control the pace may help students to overcome logistic or language obstacles in the materials. Students may need time to access the Internet, discern needed information in text, look up definitions of unfamiliar words, or translate from one language to another. Supporting teachers to start with and customize OER that support the kind of student engagement they value may broaden teachers’ knowledge of what is possible with OER, encouraging teachers as advocates in their schools or district for future selection of OER that go beyond the typical transmission-oriented approaches.

In summary, these findings support reimagining the role of OER in the science curriculum, shifting away from the use of OER for streamlining logistics or reducing costs of textbooks towards enabling teachers to leverage OER to promote knowledge integration and self-directed learning in science. As districts and schools plan for re-opening, the opportunities afforded by OERs buttress the value of ensuring that students have access to technology and accompanying curriculum materials that empower them to leverage their curiosity during science learning.

## Data Availability

Not applicable.

## Footnotes

1 In WISE, each unit has two or more explanation items scored using a natural language processing algorithm developed by partners at the Educational Testing Service called craterML©. With this algorithm, a score appears beside each student explanation in the teacher tools (Liu et al., 2016).

## Code Availability

All curriculum materials featured in this research are open-source.
